# Supplementary material for: Lipid Droplet Formation Is Regulated by Ser/Thr Phosphatase PPM1D via Dephosphorylation of Perilipin 1
Source: Int J Mol Sci. 2022 Oct 10;23(19):12046. doi: 10.3390/ijms231912046 (PMC9569567; doi:10.3390/ijms231912046)
Supplement: Supplementary file 1 [file ijms-23-12046-s001.zip › ijms-1907653-supplementary.pdf]

Supplementary Materials

Lipid Droplet Formation is Regulated by Ser/Thr Phosphatase PPM1D via Dephosphorylation of Perilipin 1

Rui Kamada [1,*,†], Sae Uno [1,†], Nozomi Kimura [1], Fumihiko Yoshimura [2], Keiji Tanino [3] and Kazuyasu Sakaguchi [1,*]

1 Laboratory of Biological Chemistry, Department of Chemistry, Faculty of Science, Hokkaido University, Sapporo 060-0810, Japan
2 School of Pharmaceutical Sciences, University of Shizuoka, Shizuoka 422-8526, Japan
3 Laboratory of Organic Chemistry II, Department of Chemistry, Faculty of Science, Hokkaido University, Sapporo 060-0810, Japan
* Correspondence: kamadar@sci.hokudai.ac.jp (R.K.); kazuyasu@sci.hokudai.ac.jp (K.S.)
† These authors contributed equally to this work.

Contents:

Figure S1. Fluorescence probe TAP-2CY4E1 specifically stained lipid droplets

Figure S2. TAP-2CY4E1 staining of PPM1D-inhibited mature 3T3-L1 adipocytes withPPM1D inhibition.

Figure S3. Western blot analysis of perilipin 1 after 7 days of treatment of SL-176 in mature3T3-L1 white adipocytes.

Figure S4. Effects of PPM1D inhibitor SL-176 on perilipin 1 mutants.

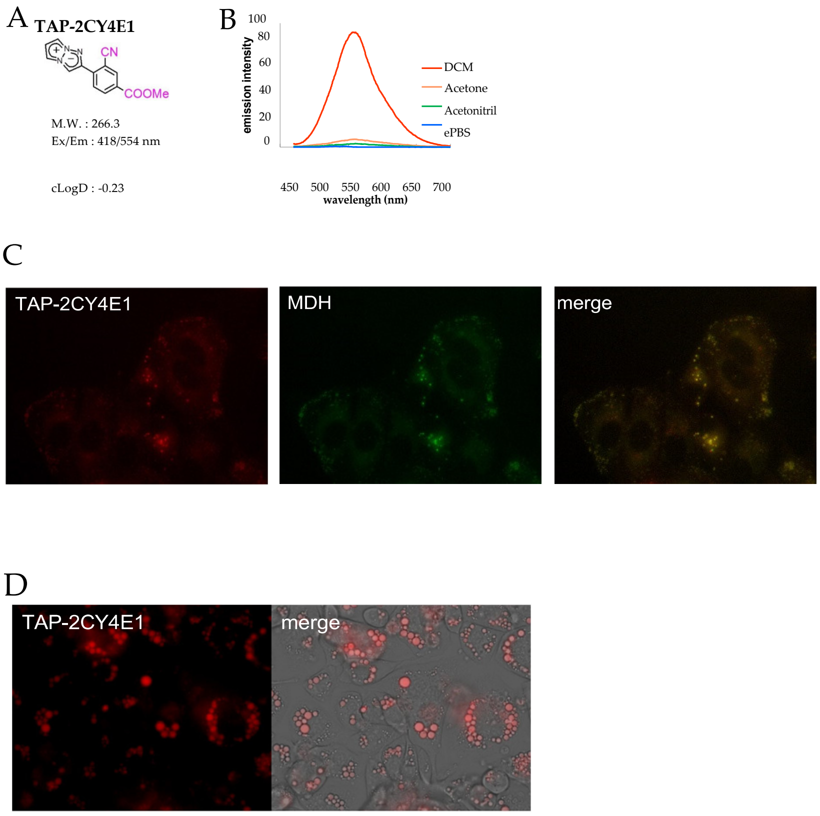

Figure S1. Fluorescence probe TAP-2CY4E1 specifically stained lipid droplets
(A) Chemical structure of the 1,3a,6a-triazapentalene derivative TAP-2CY4E1 [compound 1e, [29]]. (B) Fluorescence emission spectra of TAP-2CY4E1 (10 μM) in aqueous and organic solvents. TAP-2CY4E1 showed strong fluorescence in dichloromethane (DCM), which is a hydrophobic organic solvent. (C) Co-staining with the lipid droplet probes MDH and TAP-2CY4E1. Human lung

cancer-derived A549 cells were treated with 10 μM of TAP-2CY4E1 (red) and 10 μM MDH (green), and confocal luminescence images of living A549 cells were observed using fluorescence microscopy. TAP-2CY4E1 showed the same pattern as the lipid droplet probe MDH, showing strong fluorescent signals in lipid droplets. This result indicates that TAP-2C4E1 is a selective lipid droplet probe. (D) Fluorescent staining of differentiated 3T3-L1 adipocytes by TAP- 2CY4E1 (10 μM). "Merge" represents the merged image of TAP-2CY4E1 (TRITC filter) image and transmitted light image. This result revealed that the lipid droplets of adipocytes could be visualized using the fluorescent probe.

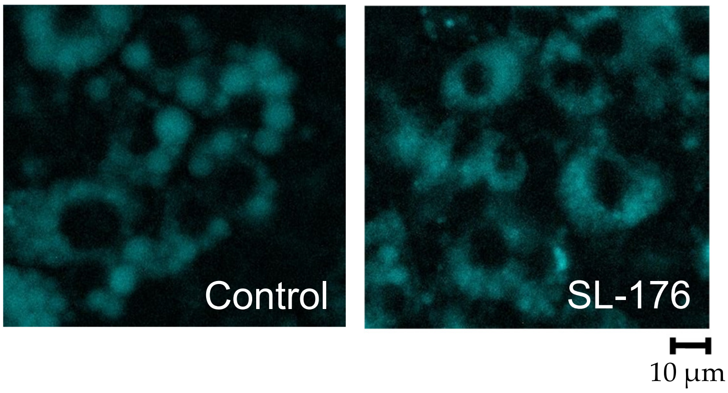

Figure S2. TAP-2CY4E1 staining of mature 3T3-L1 adipocytes with PPM1D inhibition. Lipid droplet imaging of PPM1D-inhibited mature 3T3-L1 adipocytes using TAP- 2CY4E1. After differentiation for 8 days followed by treatment with SL-176 for 7 days, the cells were stained with TAP-2CY4E1. TAP-2CY4E1 can visualize lipid droplets in living cells by simply adding a probe to the cell culture media. After observation under a fluorescent microscope, TAP-2CY4E1 was removed by washing the cells, which could then be used for another biological assay.

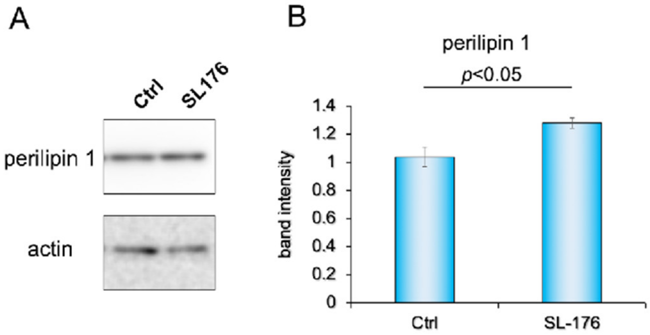

Figure S3. Western blot analysis of perilipin 1 after 7 days of treatment of SL-176 in mature 3T3-L1 white adipocytes.

(A) Representative pictures of perilipin 1 and actin expressions in the mock- and SL-176- treated mature 3T3-L1 white adipocytes for 7 days. (B) Bar graphs of perilipin 1 expression levels normalized to actin. Data represents the mean ± S.D. of three independent experiments. Significance was analyzed by using Student t-test.

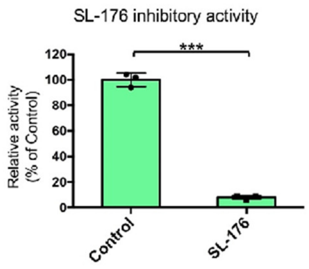

Figure S4. Effects of PPM1D inhibitor SL-176 on perilipin 1 mutants.
Inhibitory activity of the SL-176 (1 μM) against phosphatase activity of PPM1D with the phosphorylated perilipin 1 peptide. Data represents mean ± S.D. of three independent experiments. Significance was analyzed by using Student t-test. *** $p<0.001$.
